# 770 Outcome Results of the Prospective CEA Registry

**DOI:** 10.1093/jbcr/irad045.245

**Published:** 2023-05-15

**Authors:** Bounthavy Homsombath, Rajiv Sood, Brett Hartman, Shawn Fagan, John Griswold

**Affiliations:** JMS Burn Center, Augusta, Georgia; Burn and Reconstructive Centers of America, Augusta, Georgia; Indiana university/Eskenazi Health, Indianapolis, Indiana; JMS Burn Center, Augusta, Georgia; Texas Tech University Health Sciences Center, Lubbock, Texas; JMS Burn Center, Augusta, Georgia; Burn and Reconstructive Centers of America, Augusta, Georgia; Indiana university/Eskenazi Health, Indianapolis, Indiana; JMS Burn Center, Augusta, Georgia; Texas Tech University Health Sciences Center, Lubbock, Texas; JMS Burn Center, Augusta, Georgia; Burn and Reconstructive Centers of America, Augusta, Georgia; Indiana university/Eskenazi Health, Indianapolis, Indiana; JMS Burn Center, Augusta, Georgia; Texas Tech University Health Sciences Center, Lubbock, Texas; JMS Burn Center, Augusta, Georgia; Burn and Reconstructive Centers of America, Augusta, Georgia; Indiana university/Eskenazi Health, Indianapolis, Indiana; JMS Burn Center, Augusta, Georgia; Texas Tech University Health Sciences Center, Lubbock, Texas; JMS Burn Center, Augusta, Georgia; Burn and Reconstructive Centers of America, Augusta, Georgia; Indiana university/Eskenazi Health, Indianapolis, Indiana; JMS Burn Center, Augusta, Georgia; Texas Tech University Health Sciences Center, Lubbock, Texas

## Abstract

**Introduction:**

Cultured epidermal autograft (CEA) is a permanent skin replacement indicated for use in adult and pediatric patients with deep dermal or full thickness burns comprising a total body surface area (TBSA) ≥ 30%. CEA may be used in conjunction with split-thickness autografts or alone, in patients for whom split-thickness autografts may not be an option due to the severity and extent of their burns. CEA was approved for use in adults in the United States in 2007 as a Humanitarian Use Device (HUD) under a Humanitarian Device Exemption (HDE) and was approved for pediatric use in 2016.

**Methods:**

The objective of the CEA Registry is to allow prospective collection and analysis of demographic, treatment, and outcome data for the real-world use of CEA. Prior to initiation of the Registry, a central Institutional Review Board reviewed and approved written procedures and informed consent from each participating burn center. As of the date of this analysis, 5 centers were enrolling patients in the Registry. The data are collected and stored in a 21 Code of Federal Regulations (CFR) Part 11 and Health Insurance Portability and Accountability Act (HIPAA) compliant environment. First patient enrollment in the Registry was 13 June 2019 and data cutoff for this analysis was 28 July 2022.

**Results:**

Sixtyeight patients (50 adults and 18 children) had completed data in the registry, up through hospital discharge, for this analysis. Except for age and revised Baux score, demographics and burn characteristics were similar between the adult and pediatric populations. The majority of patients were male in both the adult (76%) and pediatric (67%) groups, with burn caused by flame the most common etiology (86% and 78%, respectively). The total body surface area (TBSA) of burn was 58% in adults and 56% in pediatric patients (range 30% to 96%), and almost half had inhalation injury in each group. Approximately 74% of adults and 67% of pediatric patients had TBSA ≥50%. Median age in the adult group was 42 years, and in the pediatric group was 15 years. Since age is a component of the calculation of Baux score, pediatric patients had lower mean revised Baux scores (75) compared to adults (107.5). Overall, 59 of the 68 patients (87%) survived until hospital discharge. The mean % graft take (engraftment) in both adult (81%) and pediatric (84%) groups is notable when compared to mean final graft take in 453 Epiceltreated patients reported in the HDE database (67% in adults and 70% in pediatric). By age group, adults had 84% survival rate and pediatric patients had 94% survival.

**Conclusions:**

Outcomes from this prospective collection of data in severely burned patients treated with CEA demonstrate favorable engraftment and survival rates and are in general agreement with recent literature.

**Applicability of Research to Practice:**

Reported outcomes from the CEA Registry can be used as a benchmark by centers using CEA for treatment of burns.

